# Supplementary material for: Cognitive and Interpersonal Factors in Adolescent Inpatients with Anorexia Nervosa: A Network Analysis
Source: Children (Basel). 2023 Apr 15;10(4):730. doi: 10.3390/children10040730 (PMC10137195; doi:10.3390/children10040730)
Supplement: Supplementary file 1 [file children-10-00730-s001.zip › children-2331915-supplementary.pdf]

Supplementary Materials

The EVHAN Group Members:

Christophe Lalanne2, Jeanne Duclos1,2,3, Lama Mattar1,2, Hélène Roux1,2, Marie Raphaële Thiébaud1,2, Sarah Vibert1,2, Tamara Hubert2, Annaig Courty1,4, Damien Ringuenet4, Jean-pierre Benoit1,5, Corinne Blanchet1,5, Marie Rose Moro1,5, Laura Bignami6, Clémentine Nordon6, Frédéric Rouillon6,7, Solange Cook8, Catherine Doyen6,8, Marie-Christine Mouren Siméoni6,
Priscille Gerardin9, Sylvie Lebecq9, Marc-Antoine Podlipski9, Claire Gayet9, Malaika Lasfar9, Marc Delorme10, Xavier Pommereau10, Stéphanie Bioulac10,11, Manuel Bouvard10,12, Jennifer Carrere10, Karine Doncieux13, Sophie Faucher13, Catherine Fayollet13, Amélie Prexl13, Stéphane Billard14,15, François Lang14,15, Virginie Mourier-Soleillant14, Régine Greiner14, Aurélia Gay14,15, Guy Carrot14,15, Sylvain Lambert16, Morgane Rousselet16,17, Ludovic Placé16,17, Jean-luc Venisse16,17, Marie Bronnec16,17, Bruno Falissard18, Christophe Genolini19, Christine Hassler18, Jean-Marc Tréluyer20, Olivier Chacornac20, Maryline Delattre20, Nellie Moulopo20, Christelle Turuban20 and Christelle Auger20.

1. CESP, INSERM, University Paris-Sud, UVSQ, University Paris-Saclay, Paris, France
2. Institut Mutualiste Montsouris, Paris, France
3. University of Reims, EA 6291, Reims, France
4. Hospital Paul Brousse, AP-HP, Villejuif, France
5. Maison de Solenn, Hospital Cochin, AP-HP, Paris, France
6. CMME, Saint Anne Hospital, Paris, France
7. INSERM Center 894, Paris, France
8. Hospital Robert Debré, AP-HP, Paris, France
9. University Hospital of Rouen, France
10. University Hospital of Bordeaux, Bordeaux, France
11. USR CNRS 3413 SANPSY, Bordeaux, France
12. University Victor Ségalen Bordeaux 2, Bordeaux, France
13. Institut Marcel Rivière, La Verrière, Le Mesnil Saint-Denis, France
14. University Hospital of Nord, Saint-Etienne, France
15. University of Saint-Etienne, EA 4556 laboratory Epsylon, France
16. University Hospital of Nantes, Nantes, France
17. University of Nantes, EA 4275, France
18. CESP, INSERM, University Paris-Sud, UVSQ, University Paris-Saclay, Villejuif, France
19. UMR 1027, Toulouse, France
20. URC-CIC Cochin Necker, AP-HP, Paris, France

Table S1. Goldbricker Bad Pairs

**'Bad pairs'**	**% Of different correlations**	**Resulting nodes**	**Description**
EDEQ Weight & EDEQ Shape	0	Combined	Overvaluation of weight and shape
EDEQ Shape *& EDEQ Eating*	.07		
EDEQ Weight *& EDEQ Eating*	.07		
FAD Global Functioning & ***F-MPS Parental Criticism***	.07		
EDEQ Eating & EDEQ Restraint	.13		

EDEQ Weight & *EDEQ Restraint*	.13		
F-MPS Parental Expectations & F-MPS Parental Criticism	.13	Combined	Perceived parental expectations and criticism

Figure S1. Network with BMI and FLEX

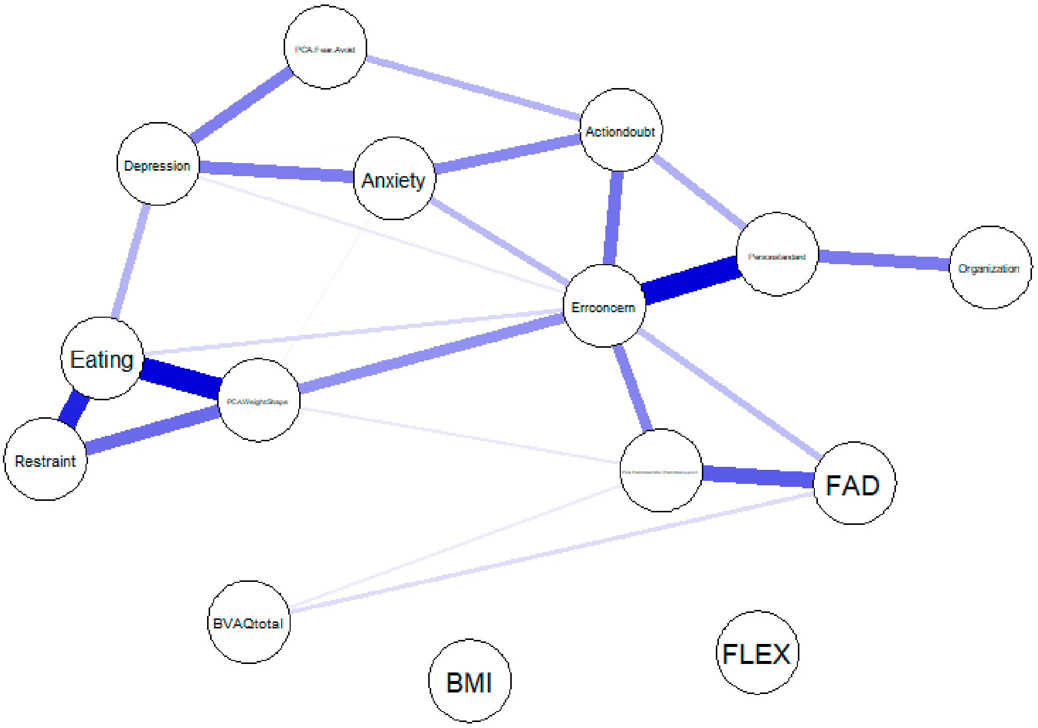

Figure S2. Mean predictability of network nodes

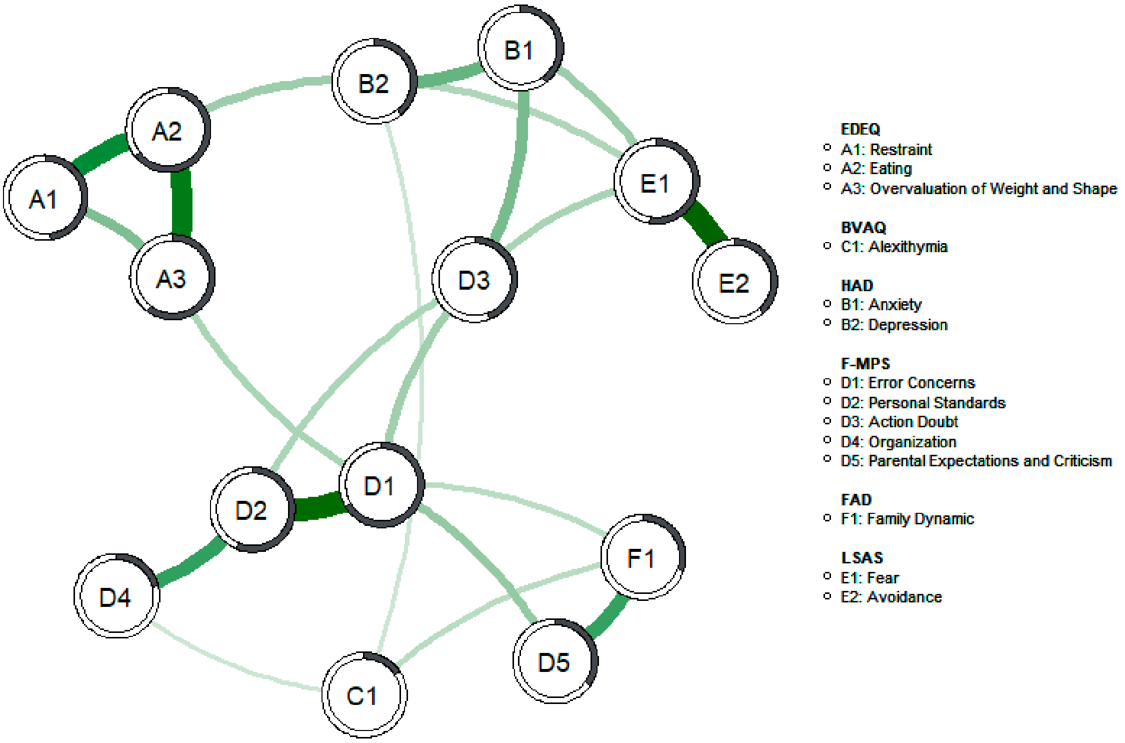

Figure S3. Bootstrapped strength and bridge strength stability

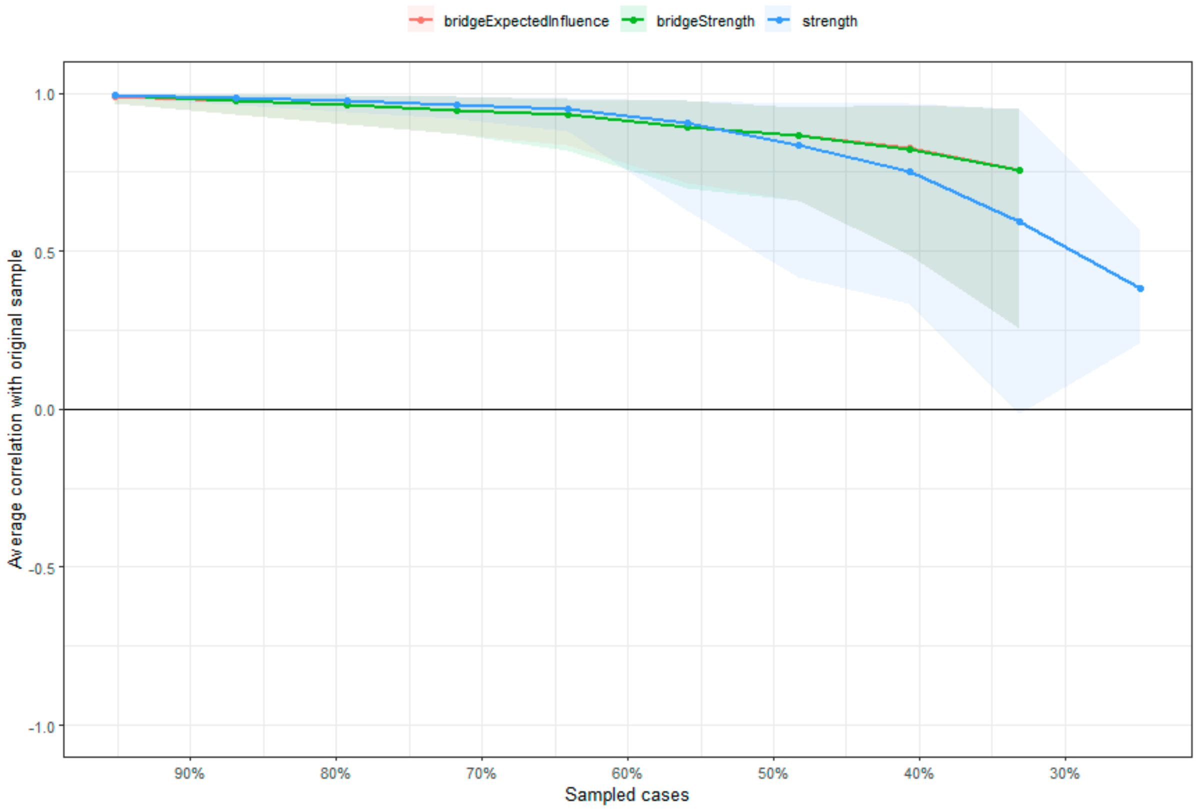

Figure S4. Bootstrapped confidence intervals of edge weights

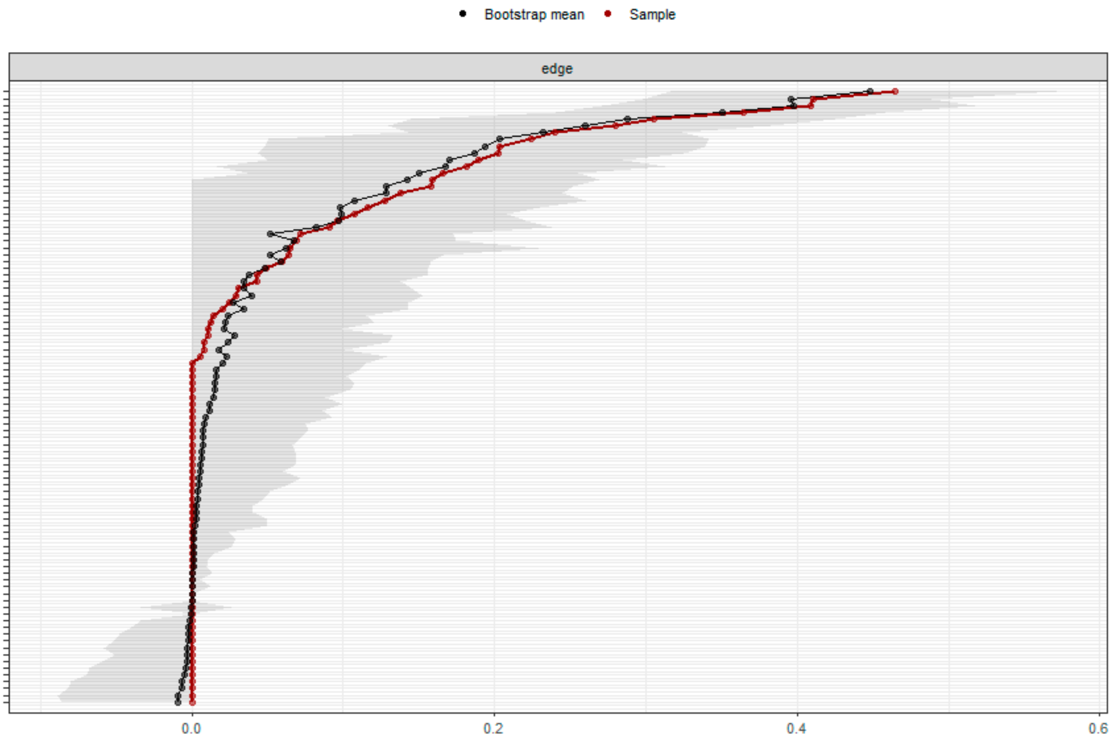

Note: grey areas represent confidence intervals

Figure S5. Results of edge difference test

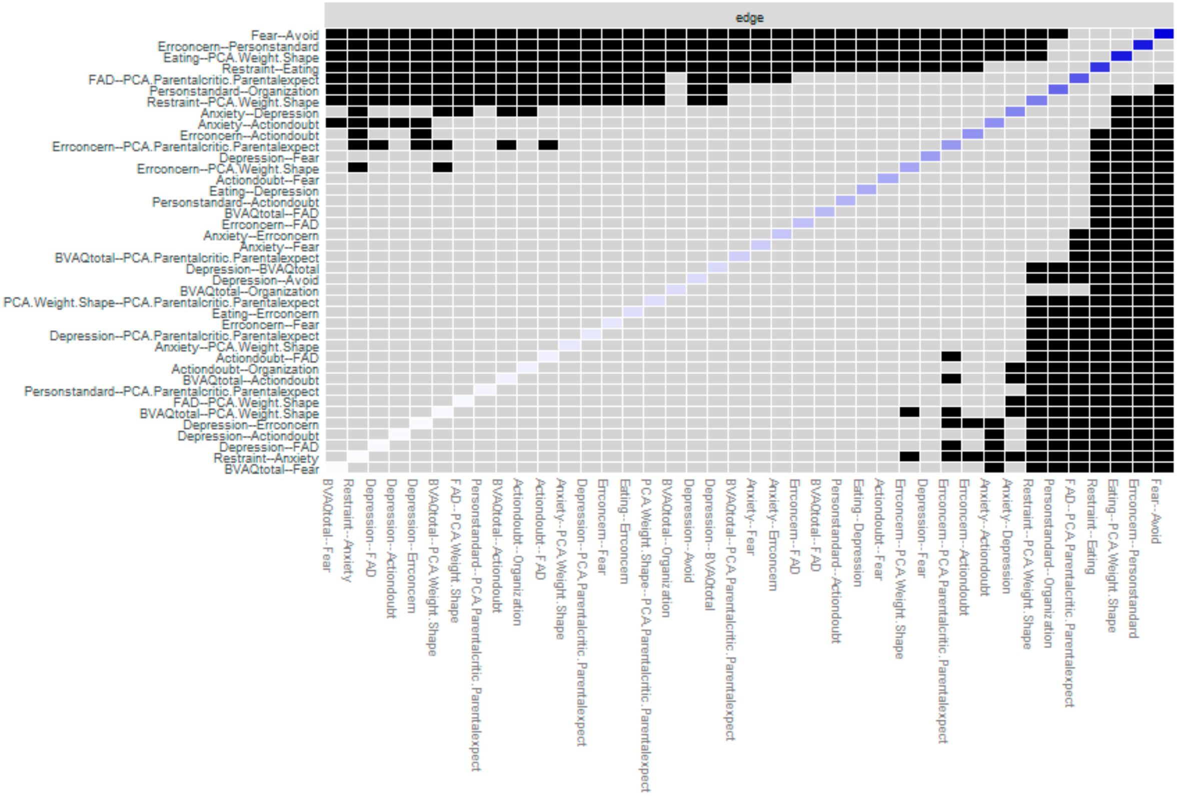

Note: Edges are presented in descending order of edge weight values. Black squares represent statistically significant differences

Figure S6. Bootstrapped difference test for strength

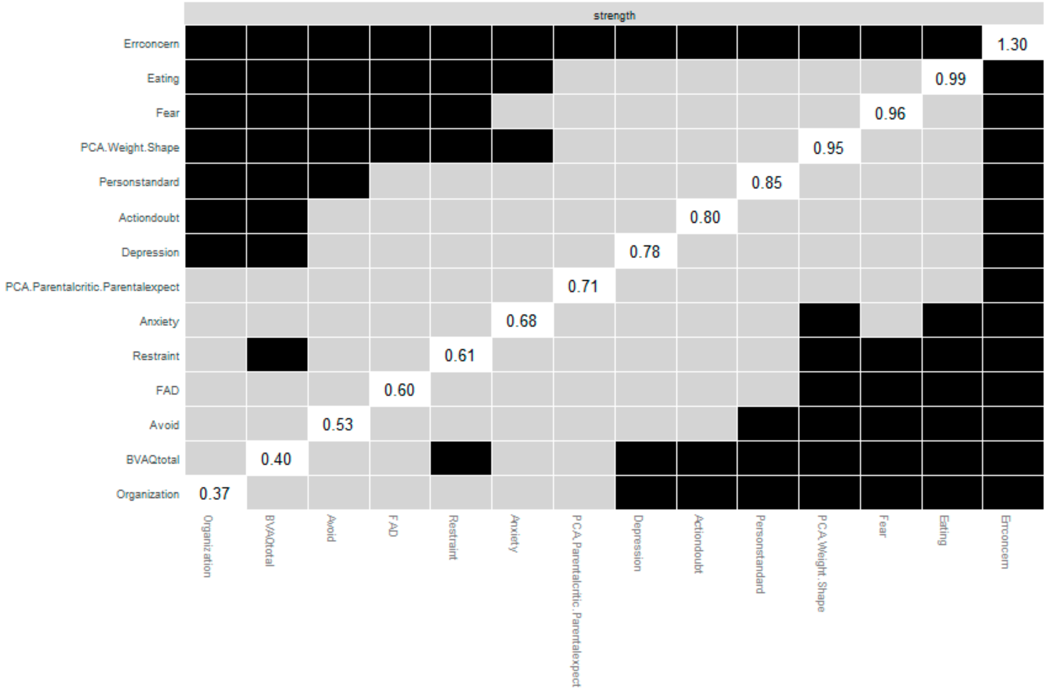

Bootstrapped difference test resuts for bridge strength. Variables are presented in descending order of strength values. Black squares represent statistically significant differences. Errconcern: Concern over Mistakes, Actiondoubt: Doubt about Actions, PCA.WeightShape:

Overvaluation of Weight and Shape, PersonStandard: Personal Standards, BVAQ total: Alexithymia total score, PCA.Parentcritic.ParentExpect: Parental Criticism and Expectations, FAD: Family Assessment Device.

Figure S7. Bootstrapped difference test for bridge strength

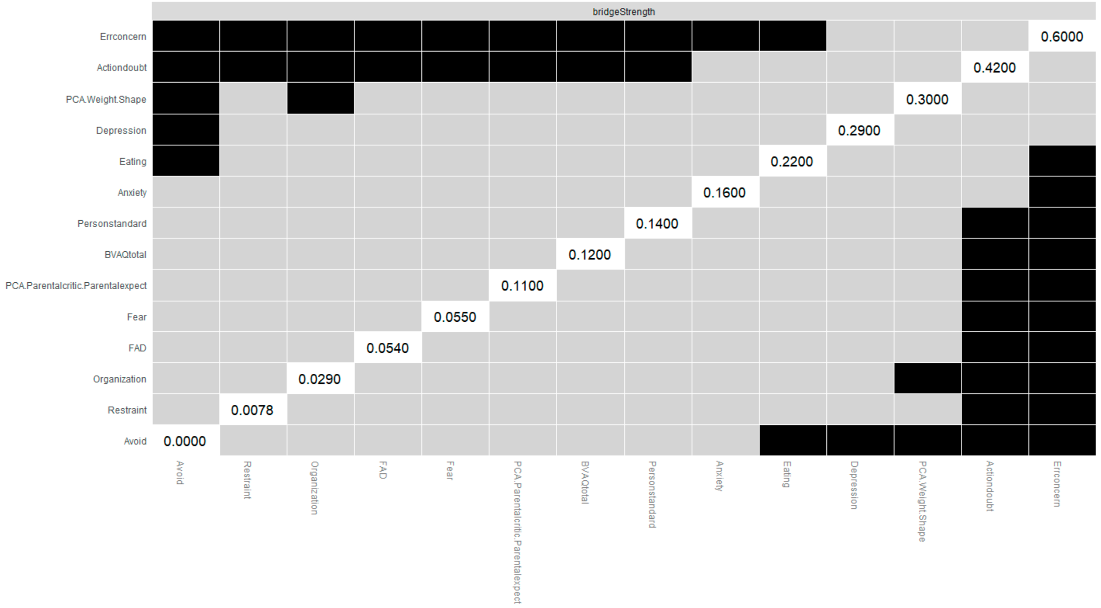

Bootstrapped difference test resuts for bridge strength. Variables are presented in descending order of strength values. Black squares represent statistically significant differences. Errconcern: Concern over Mistakes, Actiondoubt: Doubt about Actions, PCA.WeightShape: Overvaluation of Weight and Shape, PersonStandard: Personal Standards, BVAQ total: Alexithymia total score, PCA.Parentcritic.ParentExpect: Parental Criticism and Expectations, FAD: Family Assessment Device.

```
##----R CODE NETWORK ANALYSIS----##

library('dplyr')
library('magrittr')
library('glasso')
library('qgraph')
library ('bootnet')
library('readr')
library('huge')
library('MVN')
library('networktools')
library('tidyverse')
library('mgm')

##----Data Preparation ----##

data_g <-Network_BVAQ_total

#selection subsection of variables

DataSelection <- select(data_g, c(2:7, 13:24))

##Checking multivariate normality assumption##
#Mardia's test
mvn(DataSelection_s, mvnTest="mardia")

#Henze-Zirkler's test
mvn(DataSelection_s, mvnTest="hz")

#Royston's test
mvn(DataSelection_s, mvnTest="dh")
```

```
# NPN transformation and compute correlations:
data_npn_s <- huge.npn(DataSelection_s)

#goldbricker

gb_fullnetwork<- goldbricker(data_npn_s, p=0.01, threshold=0.2)

gb_fullnetwork

badpairs <- c("Weight", "Shape",
              "Parentalcritic", "Parentalexpect",
              "Fear","Avoid")

reduced_network <- net_reduce(data=data_npn, badpairs=badpairs)
reduced_network <- as.data.frame(apply(reduced_network,2,as.numeric))

##redoing data selection steps as BMI and FLEX were not connected to the network
#making bootstrapping and bridge networks impossible (See supplementary Figure 1)

DataSelection2 <- select(data_g, c(2:7, 13:22))

#NPN transformation and compute correlations:
data_npn <- huge.npn(DataSelection2)

#goldbricker

gb_fullnetwork <- goldbricker(data_npn, p=0.01, threshold=0.2)
gb_fullnetwork

badpairs <- c("Weight", "Shape",
              "Parentalcritic", "Parentalexpect")
```

```
reduced_network <- net_reduce(data=data_npn, badpairs=badpairs)

reduced_network <- as.data.frame(apply(reduced_network,2,as.numeric))

##----Network Analysis----##

graph <- estimateNetwork(reduced_network, default =
                          "EBICglasso", tuning = 0.5,
                        corMethod = "cor_auto")
plot(graph)

#comparison with Spearman correlation - ultimately not used as
##cor_auto network was not particularly dense, nor did it have unexpected negative or large edges

graph3 <- estimateNetwork(reduced_network, default =
                           "EBICglasso", tuning = 0.5,
                         corMethod = "spearman")
plot(graph3)

##bootstrapping##

boot1 <- bootnet(graph, nBoots = 2500, nCores = 2, type =
                   "nonparametric", statistics = "all", communities = c( "1", "1", "2", "2", "3", "3", "3", "2",
"3", "2", "2", "3", "1", "3"))

summary(boot1, statistics = c("edge", "strength"))
plot(boot1, statistics = "bridgeStrength",
                    plot="difference", order = "sample")

plot(boot1, statistics = "Strength",
                   plot="difference", order = "sample")

plot(boot1, order = "sample", labels = FALSE)
```

```
# Plot significant differences (alpha = 0.05) of edges:
plot(boot1, "edge", plot = "difference",onlyNonZero = TRUE,
     order = "sample")

# Plot significant differences (alpha = 0.05) of node strength:
plot(boot1, "strength", plot = "difference", order = "mean")

centralitytest1 <- differenceTest(boot1,  "Restraint", "PCA.Weight.Shape", "strength", verbose =
TRUE)
centralitytest1

centralitytest2 <- differenceTest(boot1,  "Eating", "PCA.Weight.Shape", "strength", verbose = TRUE)

save(boot1, file = "boot_edges.RData")

#case dropping bootstrap
#edge = 0.441 and strength = 0.441

boot2 <- bootnet(graph, nBoots = 2500, nCores = 2,
                 type = "case",communities = c( "1", "1", "2", "2", "3", "3", "3", "2", "3", "2", "2", "3", "1",
"3"),
                 statistics = c( "bridgeStrength"))

plot(boot2, statistics = "all")
corStability(boot2)

save(boot2, file = "boot_centrality.RData")

##----predictability----##

set.seed(1)
reduceddata_mgm <- mgm(data=reduced_network,
               type = c(rep("g", 14)),
```

```
            ruleReg = "AND",
            k = 2)

reduceddata_pred_model <- predict(object = reduceddata_mgm,
                          data = reduced_network,
                          errorCon = c("R2"))
reduceddata_x2 <- as.matrix(reduceddata_pred_model$errors)

reduceddata_error_model <- c(reduceddata_x2[1:14,2])
reduceddata_error_model <- as.numeric(reduceddata_error_model)
reduceddata_error_model <- abs(reduceddata_error_model)

mean(reduceddata_error_model)
reduceddata_error_model

error_list <- list() # List for ring-segments
for(i in 1:14) error_list[[i]] <- reduceddata_pred_model$errors[i,2]
beyondmarg <- reduceddata_pred_model$errors[14,3]-reduceddata_pred_model$errors[14,5]

set.seed(1)
network_pred <- qgraph(reduceddata_mgm$pairwise$wadj, pie = error_list,
       layout= "spring", theme="colorblind", tuning = 0.5,
      sampleSize = 145, legend.cex = 0.4, groups=group.item,
      nodeNames=Names1, labels = Labels1a,
color=c("white","white","white","white","white","white"),
      pieColor = "#46494f", label.cex = .9,
        edge.color = reduceddata_mgm$pairwise$edgecolor,
        curveAll = TRUE, curveDefault = .6,
        cut = 0)

##----Centrality Analysis----#
#creating correlation matrix and using pairwise observations
```

```
A <-cor_auto(reduced_network, missing = "pairwise")

network<-EBICglasso(S=A, n=145)

plot <- qgraph(network, layout="spring")

centrality <- centralityPlot(network, include = c("Strength")
c<- centrality_auto(network)
x <- c$node.centrality$ExpectedInfluence
var <- apply(reduced_network,2,var)
cor(var,x)
cor.test(var,x)

##----bridge analysis----##

bridge <- bridge(network, communities = null,
                 useCommunities = "all", directed = NULL, nodes = NULL)

bridge$communities
plot(bridge, include=c("Bridge Strength"), order = "value", zscore=TRUE)
bridge$`Bridge Strength`
pdf("bridgeEI.pdf",width=10)

##examining with different communities
bridge1.2 <- bridge(network, communities =c( "1", "1", "2", "2", "3", "3", "3", "2", "3", "2", "2", "3",
"1", "3"),
                useCommunities = c("1", "2"), directed = NULL, nodes = NULL)
plot(bridge1.2, include=c("Bridge Expected Influence (1-step)", "Bridge Strength"), order = "value",
zscore=TRUE)
cor.test(bridge$`Bridge Strength`,bridge$`Bridge Expected Influence (1-step)`)

##----plotting regular and community network----##
```

```
Names1<-c("Restraint", "Eating",
        "Anxiety", "Depression",
        "Alexithymia",
        "Concern over Errors", "Personal Standards",
        "Doubt about Actions", "Organization", "Fear", "Avoidance", "Family Dynamic",
        "Overvaluation of Weight and Shape",
        "Parental Expectations and Criticism")
Labels1a<-c("A1","A2","C1", "C2","B1", "D1", "D2", "D3", "D4", "F1", "F2", "E1","A3", "D5" )
group.item <- list("EDEQ" = c(1,2,13),"BVAQ" = 5,"HAD" = 3:4,"F-MPS" = c (6:9, 14),"FAD" = 12,
"LSAS" = 10:11)
network_colour <-EBICglasso (S=A, n=145)
plot_colour <- qgraph (network_colour, layout = "spring", groups=group.item, tuning = 0.5,
                    sampleSize = 145, legend.cex = 0.6, groups=group.item,
                    nodeNames=Names1, labels = Labels1a,
                    palette = 'pastel', posCol = "#003399",
                    negCol = "#FF9933", color = c('magenta', 'yellow', 'pink', 'aquamarine',
                                        'green', 'purple'), borders = FALSE, vTrans = 300)


#Community graph


Bridge_network <- qgraph(network_colour,
                  layout = "spring", tuning = 0.5,
                  sampleSize = 145, legend.cex = 0.6, groups=c( "1", "1", "2", "2", "3", "3", "3", "2",
"3", "2", "2", "3", "1", "3"),
                  nodeNames=Names1, labels = Labels1a,
                  palette = 'pastel', posCol = "#003399",
                  negCol = "#FF9933", color = c('magenta', 'yellow',  'aquamarine'), borders = FALSE,
vTrans = 300)
```
